# Differentially Expressed Genes in Osteomyelitis Induced by *Staphylococcus aureus* Infection

**DOI:** 10.3389/fmicb.2018.01093

**Published:** 2018-05-25

**Authors:** Peisheng Chen, Zilong Yao, Ganming Deng, Yilong Hou, Siwei Chen, Yanjun Hu, Bin Yu

**Affiliations:** ^1^Department of Orthopaedics and Traumatology, Nanfang Hospital, Southern Medical University, Guangzhou, China; ^2^Guangdong Provincial Key Laboratory of Bone and Cartilage Regenerative Medicine, Nanfang Hospital, Southern Medical University, Guangzhou, China

**Keywords:** osteomyelitis (OM), *Staphylococcus aureus* (*S. aureus*), candidate biomarkers, bioinformatic analysis, differentially expressed genes (DEGs)

## Abstract

Osteomyelitis (OM) is a complicated and serious disease and its underlying molecular signatures of disease initiation and progression remain unclear. *Staphylococcus aureus (S. aureus)* is the most common causative agent of OM. Previous study of Banchereau et al. has established a link between whole blood transcription profiles and clinical manifestations in patients infected with *S. aureus*. However, the differentially expressed genes (DEGs) in OM induced by *S. aureus* infection have not been intensively investigated. In this study, we downloaded the gene expression profile dataset GSE30119 from Gene Expression Omnibus, and performed bioinformatic analysis to identify DEGs in *S. aureus* infection induced OM from the transcriptional level. The study consisted of 143 whole blood samples, including 44 healthy controls, 42 OM-free, and 57 OM infection patients. A total of 209 *S. aureus* infection-related genes (SARGs) and 377 OM-related genes (OMRGs) were identified. The SARGs were primarily involved in the immune response by GO functional and pathway enrichment analysis. Several proteins adhere to neutrophil extracellular traps may be critical for the immune response to the process of *S. aureus* infection. By contrast, the OMRGs differ from the SARGs. The OMRGs were enriched in transmembrane signaling receptor and calcium channel activity, cilium morphogenesis, chromatin silencing, even multicellular organism development. Several key proteins, including PHLPP2 and EGF, were hub nodes in protein–protein interaction network of the OMRGs. In addition, alcoholism, systemic lupus erythematosus and proteoglycans in cancer were the top pathways influenced by the OMRGs associated with OM. Thus, this study has further explored the DEGs and their biological functions associated with *S. aureus* infection and OM, comparing with the previous study, and may light the further insight into the underlying molecular mechanisms and the potential critical biomarkers in OM development.

## Introduction

Osteomyelitis (OM) is a bacterial infection of the bone or bone marrow, which can be due to hematogenous seeding, contiguous spread from adjacent contaminated soft tissues and joints, or direct inoculation from traumatic wounds or surgery (Lew and Waldvogel, 2004). It is an infectious disease that is difficult to diagnose, and the treatment is complex because of its heterogeneity, pathophysiology, clinical presentation, and management (Trampuz and Zimmerli, 2008; Jiang et al., 2017). In the United States, the incidence of OM during the first decade of this century was twice that of 40 years ago (Kremers et al., 2015). Additionally, recent changes in the epidemiology, pathogenesis, diagnosis, treatment, and prognosis of OM render it to be a considerable public health problem with a social impact in China (Jiang et al., 2015; Wang X. et al., 2017). Historically, haematogenous OM is seen mostly in prepubertal children and in elderly patients and can develop into a devastating or even fatal disease with a high rate of sequelae (Lew and Waldvogel, 2004; Zeller et al., 2008; Peltola and Paakkonen, 2014).

Among the pathogenic microorganisms to OM, *Staphylococcus aureus* (*S. aureus*) is the most prevalent bacterial species (Mruk and Record, 2012; Kremers et al., 2015). In the case of implant infections, *S. aureus* can form biofilms, which act as a diffusion barrier to slow down the penetration of antimicrobial agents and nutrients (Costerton et al., 2003). Chronic biofilm-based infections may be protected from host immune responses despite a sustained presence of inflammatory reaction (Wilkins et al., 2014). To solve this problem, in addition to debridement and prolonged antibiotic treatment, alternative preventive and curative approaches are currently under investigation, including surface coating with antimicrobial peptides, quorum-sensing inhibitors, biofilm-degrading enzymes, and various nanoparticles (Zimmerli, 2014; Algburi et al., 2017). Though originally viewed as an extracellular pathogen, it has been reported that *S. aureus* can survive phagocytosis by neutrophils and macrophages and even was shown to be internalized by a variety of otherwise non-phagocytic cells, such as epithelial cells, endothelial cells, fibroblasts, and osteoblasts (Strobel et al., 2016). Eventually, internalized *S. aureus* may switch to small colony variants, which persists inside host cells and helps avoiding the activation of the host immune system. This most likely represents an important immune escape strategy (Proctor et al., 2006; Josse et al., 2015). *S. aureus* internalized by osteoblasts may lead to chronic and recurrent OM (Kalinka et al., 2014; Tuchscherr et al., 2016). Internalization and intracellular survival of *S. aureus* remind that some molecules or intrinsic factors may play crucial roles in the immune response to infectious OM. Thus, a greater understanding of the bacterial pathologic mechanisms involved in the development and progression of OM is required.

The emergence of high-throughput technologies has given us the possibilities to systematically survey and explore the underlying biological system. Gene expression data from public archives are now widely used in research (Rung and Brazma, 2013). Given the promising clinical applications, such as early diagnosis, grading, and prognosis prediction for many diseases, it will be important to analyze gene expressions on the high-throughput platforms, such as microarrays (Kulasingam and Diamandis, 2008). Banchereau et al. applied a module-level analysis framework based on microarray and confirmed the association between whole blood transcriptional profiles and acute community-associated *S. aureus* infections in pediatric patients (Banchereau et al., 2012). However, the differentially expressed genes (DEGs) in OM induced by *S. aureus* infection have not been intensively investigated. In the present study, we downloaded dataset GSE30119 from Gene Expression Omnibus (GEO), and used GEO2R online tool to discover the DEGs. Furthermore, the analysis of Gene Ontology (GO) term enrichment and Kyoto Encyclopedia of Genes and Genomes (KEGG) pathways of all DEGs were performed. In addition, the integrated protein–protein interaction (PPI) network of the DEGs was constructed and the top 3 modules were extracted and analyzed. As a result, we sought to explore the DEGs and their related functions in OM induced by *S. aureus* infection from the perspective of the patients' gene transcriptional level.

## Materials and methods

### Expression profile data

**Figure 1:**
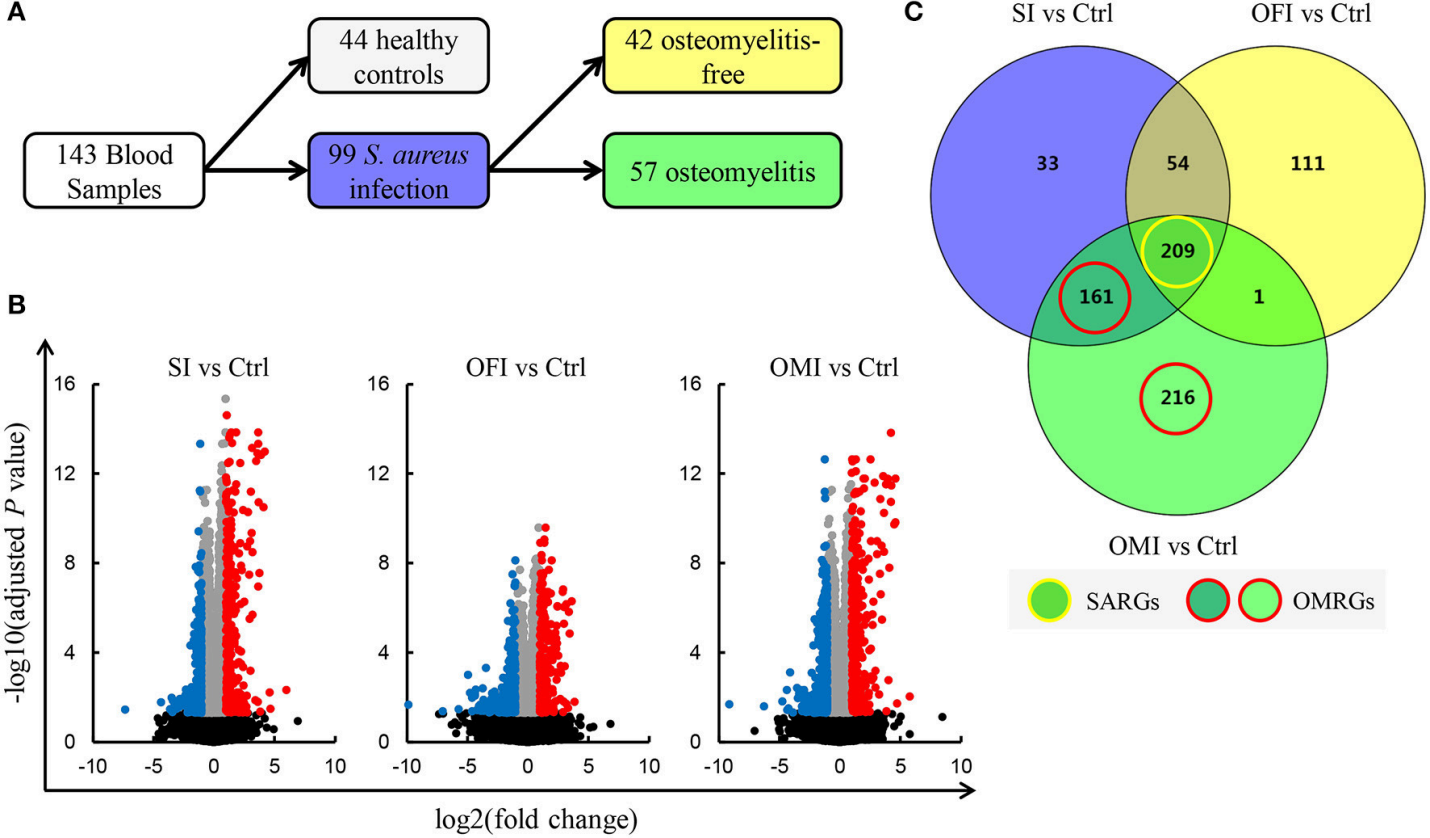
Identification of DEGs. **(A)** Blood samples schematic. **(B)** Volcano plots for DEGs. Plots of –log10(adjusted *P*-value) vs. log2(fold change) for SI vs. Ctrl, OFI vs. Ctrl, and OMI vs. Ctrl. Red plots represent the upregulated genes and blue plots represent the downregulated genes. **(C)** Venn diagram of DEGs, SARGs, and OMRGs. Yellow circle means the SARGs and red circle means the OMRGs.

The raw gene expression profile dataset GSE30119 (Banchereau et al., 2012) was downloaded from GEO (http://www.ncbi.nlm.nih.gov/geo/) (Barrett et al., 2013), which was based on the platform of GPL6947 Illumina Human HT-12 V3.0 expression beadchip. The Human HT-12 V3.0 expression beadchip features up-to-date content derived from the NCBI RefSeq database (Build 36.2, Release 22). The probe coordinates were converted from NCBI36/hg18 to GRCh38/hg38. A total of 143 whole blood samples [99 patients with community-acquired *S. aureus* infection (SI) and 44 healthy controls (Ctrl)] were included in this study. Total RNA extracted from whole blood was utilized for gene expression microarrays. The infected patients were divided into two groups, including 57 patients with OM infection (OMI) and 42 patients with OM-free infection (OFI) (Figure 1A).

### Data processing of DEGs

Banchereau et al. have established that whole blood transcriptional profiles in infected patients compared with healthy controls may reflect the variation of clinical disease manifestations (Banchereau et al., 2012). Based on their finding, we further explored the DEGs in *S. aureus* infected patients, especially those with OM, which differed from healthy controls. Significant value for DEGs in the three comparison groups, SI vs. Ctrl, OFI vs. Ctrl, and OMI vs. Ctrl, were analyzed with GEO2R (https://www.ncbi.nlm.nih.gov/geo/geo2r/) (Davis and Meltzer, 2007). The adjusted *P*-values were used to decrease the false positive rate using Benjamini and Hochberg false discovery rate method by default. Subsequently, the log2(fold change) was calculated. An adjusted *P*-value < 0.05 and |log2(fold change)| ≥ 1 were selected as threshold value for DEG screening.

### Venn diagram analysis of DEGs

Venny (http://bioinfogp.cnb.csic.es/tools/venny/index.html) was used to analyze Venn diagram for DEGs of the above three comparison groups, and the similarities and differences in three comparison groups were observed. The DEGs that overlap the three comparison groups were defined as *S. aureus* infection-related genes (SARGs). The other DEGs, observed between OMI vs. Ctrl but not OFI vs. Ctrl, were defined as OM-related genes (OMRGs).

### GO term and KEGG pathway enrichment analysis of DEGs

For high-throughput genome or transcriptome data, GO terms, including biological process (BP), cellular component (CC), and molecular function (MF), have been extensively investigated for annotating genes and identifying characteristic biological attributes (Ashburner et al., 2000). KEGG is a database used for putting associated genes into the corresponding pathways (Kanehisa et al., 2012). The Database for Annotation, Visualization and Integrated Discovery (DAVID) database (https://david.ncifcrf.gov/) was introduced to manually realize GO terms and KEGG pathway enrichment analysis of DEGs (Huang da et al., 2009). The *P*-value <0.05 was considered to have statistical significance and to achieve significant enrichment.

### PPI network and module analysis

PPI network can help us identify the key proteins and important protein modules involved in OM development in term of biological interaction. In the present study, the probe number of DEGs was converted into protein, and the co-expression graph of PPI network was obtained with the help of STRING database (http://www.string-db.org/) (Szklarczyk et al., 2015). In addition, the network was created and visualized using Cytoscape (http://www.cytoscape.org/) (Shannon et al., 2003), based on the PPIs information. After that, the topological metrics such as degree of distribution, betweenness centrality, and closeness centrality were analyzed using Cytoscape software. Nodes with higher degrees based on the number of edges (interactions) between various nodes (proteins), were regarded as hub proteins, which play key roles in the PPI network. The top 15 hub proteins with high degree of connectivity were selected. Moreover, we performed module analysis with the MCODE plugin of Cytoscape under the following thresholds of degree cutoff = 2, node score cutoff = 0.2, k-core ≥ 2, and max. depth = 100 (Bader and Hogue, 2003).

## Results

### Identification of DEGs

In the present study, a total number of 785 DEGs (Supplementary Table 1), among them 376 up-regulated and 409 down-regulated, were screened out in all three comparison groups (Figures 1B,C). It was determined that 209 SARGs present identical expression trends (Figure 1C). In addition, a total of 587 DEGs were detected after the analysis of OMI vs. Ctrl, consisting of 377 OMRGs.

### GO function enrichment analysis

To get further insight into the biological functions of identified DEGs, GO terms, and pathways enrichment analysis were investigated using DAVID. GO analysis results showed that BP of the SARGs were enriched in innate immune response, inflammatory response, proteolysis and cell adhesion, while for OMRGs including multicellular organism development, blood coagulation, calcium ion transmembrane transport, cilium morphogenesis, and chromatin silencing (Table 1). For CC, the SARGs were enriched in membrane and particular extracellular region part, such as extracellular exosome and extracellular space. By contrast, the OMRGs were mainly associated with component of membrane and plasma membrane (Table 1). In addition, MF analysis displayed that the SARGs were significantly enriched in calcium ion, carbohydrate, heparin binding, and serine-type endopeptidase and peptidase activity, and the OMRGs were enriched in phosphatidylserine binding, transmembrane signaling receptor activity, and voltage-gated calcium channel activity (Table 1; Supplementary Table 2).

**Table 1:** Gene ontology analysis of the SARGs and OMRGs (the top 5 GO terms).

**DEGs**	**Category**	**GO ID**	**Term**	**Count**	**%**	***P*-value**
SARGs	BP	GO:0045087	Innate immune response	27	12.86	1.61E-12
	BP	GO:0006955	Immune response	23	10.95	1.50E-09
	BP	GO:0006954	Inflammatory response	16	7.62	2.02E-05
	BP	GO:0006508	Proteolysis	16	7.62	0.000438
	BP	GO:0007155	Cell adhesion	14	6.67	0.001762
	CC	GO:0005886	Plasma membrane	70	33.33	1.77E-05
	CC	GO:0016021	Integral component of membrane	70	33.33	0.012861
	CC	GO:0070062	Extracellular exosome	60	28.57	6.39E-08
	CC	GO:0005615	Extracellular space	50	23.81	7.57E-15
	CC	GO:0005576	Extracellular region	40	19.05	6.59E-07
	MF	GO:0005509	Calcium ion binding	15	7.14	0.016534
	MF	GO:0004252	Serine-type endopeptidase activity	13	6.19	1.37E-05
	MF	GO:0030246	Carbohydrate binding	10	4.76	0.000201
	MF	GO:0008201	Heparin binding	7	3.33	0.006323
	MF	GO:0008233	Peptidase activity	5	2.38	0.014025
OMRGs	BP	GO:0007275	Multicellular organism development	17	4.52	0.045763
	BP	GO:0007596	Blood coagulation	9	2.39	0.026141
	BP	GO:0070588	Calcium ion transmembrane transport	7	1.86	0.027503
	BP	GO:0060271	Cilium morphogenesis	7	1.86	0.047785
	BP	GO:0006342	Chromatin silencing	6	1.6	0.00166
	CC	GO:0016021	Integral component of membrane	112	29.79	0.043901
	CC	GO:0005886	Plasma membrane	107	28.46	0.000148
	CC	GO:0070062	Extracellular exosome	73	19.41	0.002694
	CC	GO:0005887	Integral component of plasma membrane	41	10.9	0.005552
	CC	GO:0005794	Golgi apparatus	26	6.91	0.019927
	MF	GO:0004872	Receptor activity	11	2.93	0.007244
	MF	GO:0004888	Transmembrane signaling receptor activity	9	2.39	0.045717
	MF	GO:0001786	Phosphatidylserine binding	4	1.06	0.030586
	MF	GO:0005245	Voltage-gated calcium channel activity	4	1.06	0.037363

*GO, Gene Ontology; BP, biological process; CC, cell component; MF, molecular function*.

### KEGG pathway enrichment analysis

The top pathways of the SARGs and OMRGs analyzed by KEGG analysis are shown in Table 2, Supplementary Table 3. The SARGs were enriched in osteoclast differentiation, hematopoietic cell lineage, transcriptional misregulation in cancer, complement and coagulation cascades, and antigen processing and presentation, while the OMRGs were enriched in alcoholism, systemic lupus erythematosus, proteoglycans in cancer, arrhythmogenic right ventricular cardiomyopathy.

**Table 2:** KEGG pathway enrichment analysis of the SARGs and OMRGs (the top pathway terms).

**DEGs**	**Pathway ID**	**Name**	**Count**	**%**	***P*-value**
SARGs	hsa04380	Osteoclast differentiation	10	4.76	3.22E-05
	hsa04640	Hematopoietic cell lineage	8	3.81	8.14E-05
	hsa05202	Transcriptional misregulation in cancer	8	3.81	0.004749631
	hsa04610	Complement and coagulation cascades	6	2.86	0.001597673
	hsa04612	Antigen processing and presentation	6	2.86	0.002453934
	hsa04664	Fc epsilon RI signaling pathway	5	2.38	0.009949792
	hsa05150	*Staphylococcus aureus* infection	4	1.9	0.029278407
	hsa05134	Legionellosis	4	1.9	0.029278407
OMRGs	hsa05034	Alcoholism	10	2.66	0.005412177
	hsa05322	Systemic lupus erythematosus	9	2.39	0.00329809
	hsa05205	Proteoglycans in cancer	9	2.39	0.031838957
	hsa05412	Arrhythmogenic right ventricular cardiomyopathy	5	1.33	0.042148285

### Hub proteins and modules screening of the PPI network

**Figure 2:**
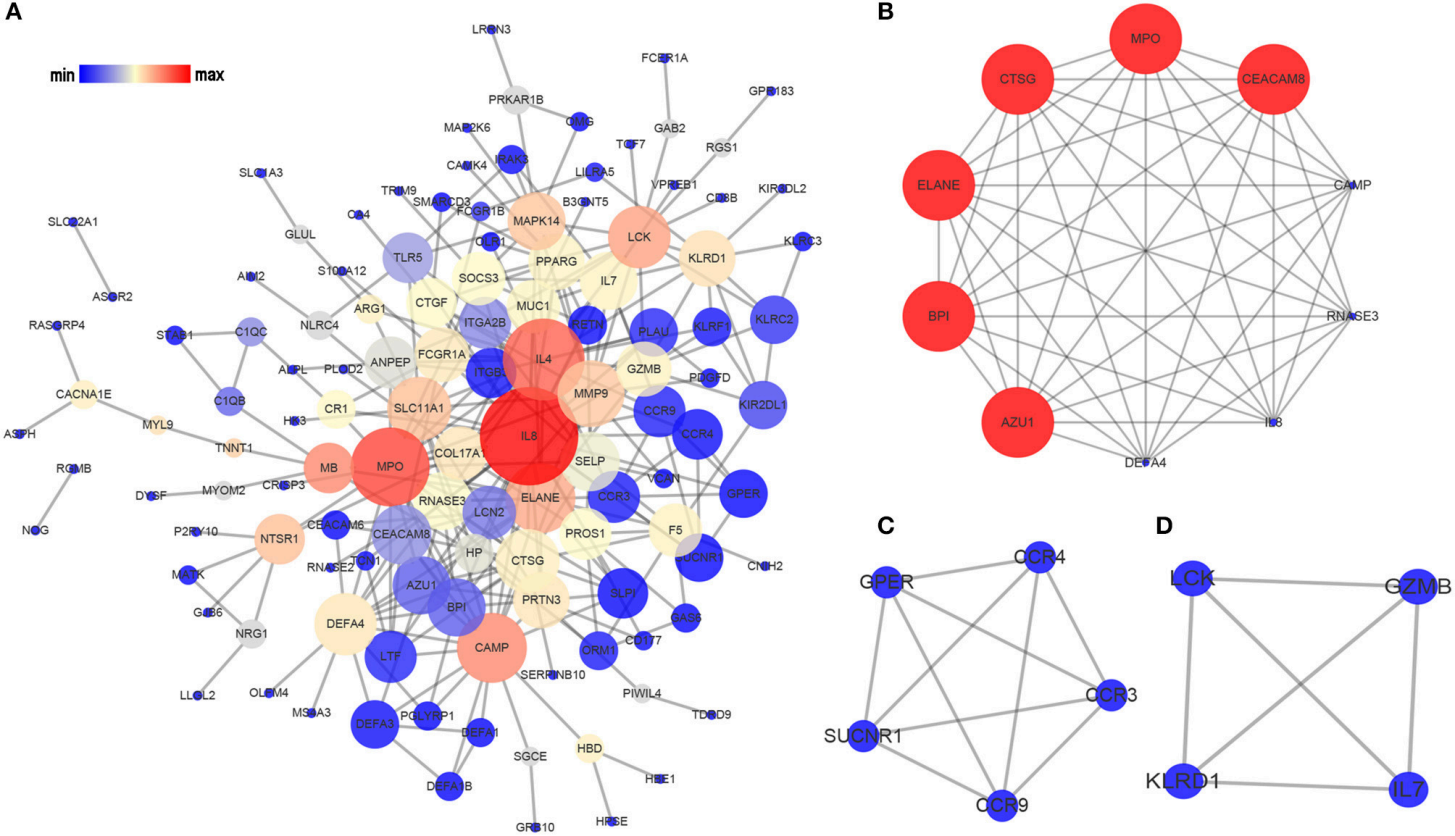
Analysis of the PPI network of the SARGs. The nodes represent the SARGs in the PPI network, and the lines show the interaction between the SARGs. **(A)** PPI network of the SARGs. **(B)** module 1. **(C)** module 2. **(D)** module 3. The node size correspond to degree of the node, while the node color denotes betweenness centrality.

Based on the information in STRING database, we made the PPI network of the DEGs. The PPI network of the SARGs was constructed with 123 nodes and 312 edges (Figure 2A; Supplementary Table 4). The results of topological analysis showed that interleukin 8 (IL8), interleukin 4 (IL4), and myeloperoxidase (MPO) were the top 3 hub proteins with higher node degrees (Table 3). In order to screen significant modules in this PPI network, we used the plugin MCODE in Cytoscape. Top 3 modules were selected (Figures 2B–D). We found that most of the proteins enriched in these 3 modules were up-regulated. Particularly, the top 5 proteins with higher node degrees in module 1 were interleukin 8 (IL8, degree = 8), myeloperoxidase (MPO, degree = 9), elastase, neutrophil expressed (ELANE, degree = 9), cathelicidin antimicrobial peptide (CAMP, degree = 8), cathepsin G (CTSG, degree = 9) (Figure 2B). The 5 nodes (all degree = 4) in module 2 were C-C motif chemokine receptor 9 (CCR9), C-C motif chemokine receptor 3 (CCR3), C-C motif chemokine receptor 4 (CCR4), G protein-coupled estrogen receptor (GPER), and succinate receptor (SUCNR) (Figure 2C). Additionally, the 4 nodes (all degree = 3) in module 3 were LCK proto-oncogene, Src family tyrosine kinase (LCK), interleukin 7 (IL7), killer cell lectin like receptor D1 (KLRD1), granzyme B (GZMB) (Figure 2D).

**Table 3:** The top 15 hub proteins identified in topological analysis of the PPI network of the SARGs and OMRGs.

**DEGs**	**Degree**	**Betweenness centrality**	**Closeness centrality**	**Gene name**	**Expression change**
SARGs	38	0.26824799	0.47773279	IL8	Down
	27	0.17520903	0.4469697	IL4	Down
	25	0.2014464	0.45559846	MPO	Up
	19	0.10405493	0.43382353	ELANE	Up
	19	0.13489376	0.3973064	CAMP	Up
	17	0.07247173	0.41403509	MMP9	Up
	15	0.03397526	0.39464883	CTSG	Up
	15	0.08304961	0.41403509	SLC11A1	Up
	14	0.11008923	0.34911243	LCK	Down
	14	0.04295219	0.35014837	DEFA4	Up
	12	0.01906122	0.38688525	SELP	Up
	12	0.01119682	0.37820513	CEACAM8	Up
	11	0.0757675	0.37341772	MAPK14	Up
	11	0.03156578	0.36990596	IL7	Down
	11	0.00741906	0.37699681	AZU1	Up
OMRGs	33	0.34532099	0.37587007	PHLPP2	Down
	23	0.28324927	0.36986301	EGF	Up
	16	0.14451349	0.36	PIKFYVE	Down
	15	0.19939732	0.38297872	HSPA8	Down
	13	0.06895809	0.30975143	HIST2H2BE	Up
	13	0.09823181	0.3375	UBE2D1	Up
	12	0.12053919	0.30740038	CD79A	Down
	11	0.0633676	0.31702544	ERBB2	Down
	11	0.04845481	0.29944547	CD86	Down
	10	0.08593619	0.33128834	MPP6	Down
	10	0.10468113	0.3246493	PCSK6	Up
	9	0.07043681	0.29294756	VWF	Up
	9	0.06127452	0.31764706	GNAO1	Up
	9	0.08899187	0.33264887	TLR2	Up
	8	0.00375522	0.26821192	HIST2H2AC	Up

**Figure 3:**
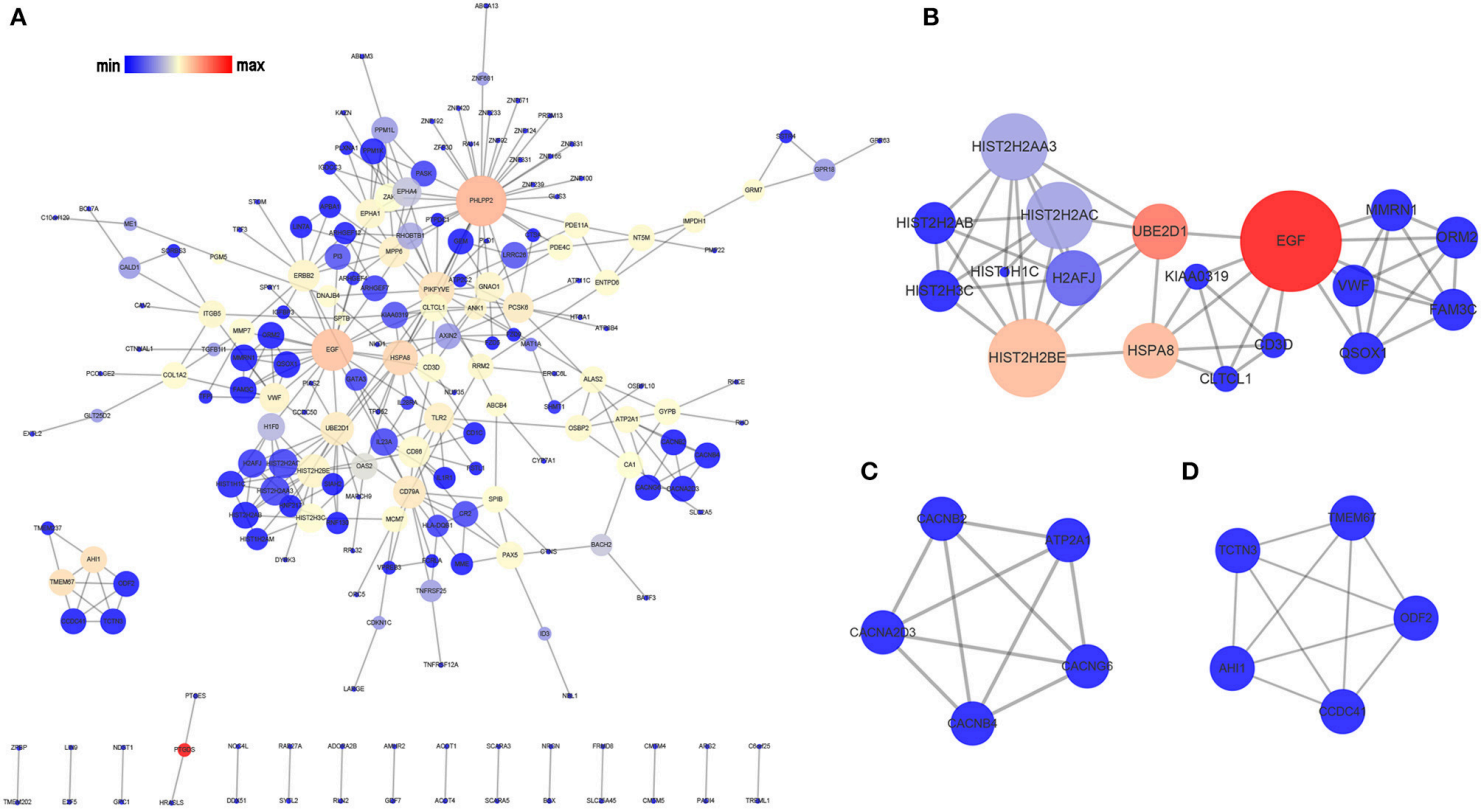
Analysis of the PPI network of the OMRGs. The nodes represent the OMRGs in the PPI network, and the lines show the interaction between the OMRGs**. (A)** PPI network of the OMRGs. **(B)** module 1. **(C)** module 2. **(D)** module 3. The node size correspond to degree of the node, while the node color denotes betweenness centrality.

The PPI network of the OMRGs consisted of 200 nodes and 336 edges (Figure 3A; Supplementary Table 5). Besides, PH domain and leucine rich repeat protein phosphatase 2 (PHLPP2), and epidermal growth factor (EGF) were the top 2 hub proteins (Table 3). The top 5 proteins with higher node degrees in module 1 were epidermal growth factor (EGF, degree = 10), Heat shock 70 kDa protein 8 (HSPA8, degree = 6), histone cluster 2, H2be (HIST2H2BE, degree = 8), ubiquitin conjugating enzyme E2 D1 (UBE2D1, degree = 6), von Willebrand factor (VWF, degree = 5) (Figure 3B). The degree of all nodes were equal to 4 in module 2 and module 3. Module 2 was composed of ATPase sarcoplasmic/endoplasmic reticulum Ca^2+^ transporting 1 (ATP2A1), calcium voltage-gated channel auxiliary subunit beta 2 (CACNB2), calcium voltage-gated channel auxiliary subunit beta 4 (CACNB4), calcium voltage-gated channel auxiliary subunit alpha2delta 3 (CACNA2D3), calcium voltage-gated channel auxiliary subunit gamma 6 (CACNG6) (Figure 3C). Module 3 contained Abelson helper integration site 1 (AHI1), transmembrane protein 67 (TMEM67), coiled-coil domain containing 41 (CCDC41), tectonic family member 3 (TCTN3), outer dense fiber of sperm tails 2 (ODF2) (Figure 3D).

## Discussion

OM has been demonstrated to be a complicated and serious disease and its most common pathogenic bacteria is *S. aureus* (Lew and Waldvogel, 2004; Kremers et al., 2015). It is of utmost importance to obtain a correct and timely diagnosis for the long-term outcomes of OM. Despite recent scientific achievements revealing that single nucleotide polymorphisms (SNPs) of several specific genes, such as vitamin D receptor (VDR) and Cyclooxygenase-2 (COX-2), may contribute to the increased susceptibility to OM (Jiang et al., 2016; Wang L. et al., 2017), the exact molecular pathophysiology of OM is still largely unknown. Moreover, individual OM-specific biomarkers within the context of *S. aureus* infection have been difficult to derive with current clinical technology. Here, we reported for the first time that identifying the DEGs and activated signaling pathways in association with *S. aureus* infection induced OM by an integrated bioinformatic analysis method.

Previous studies have proved that host blood transcriptional signatures discriminate patients with different infectious diseases, such as viral and bacterial infection (Ramilo et al., 2007; Banchereau et al., 2012; Hu et al., 2013). Interestingly, Banchereau et al. correlated transcriptome-wide expression levels with the heterogeneous clinical manifestations of infected children (Banchereau et al., 2012). In their study, 99 patients and 44 healthy controls were included. An analytical framework of 62 transcriptional modules and modular fingerprints combined with the molecular distance to health (MDTH) were applied to assess the correlation between biological function and clinical disease manifestations. Independent training (40 patients, 22 healthy controls) and test sets (59 patients, 22 healthy controls) yielded similar modular fingerprints of *S. aureus* infection as compared to healthy controls. Additionally, pathway enrichment analysis revealed blood coagulation over-expressed in patients with osteoarticular infections (Banchereau et al., 2012). However, the DEGs in OM induced by *S. aureus* infection have not been intensively explored.

To better understand the whole blood response to OM, 99 patients were assigned to two groups according to the occurrence of OM, including 42 OFI and 57 OMI patients in our study. In a similar manner to that of previous investigators (Banchereau et al., 2012), we have identified 785 DEGs of *S. aureus* infection that differed from healthy controls in all three comparison groups. Subsequently, GO and KEGG enrichment analysis were introduced to further analyze the biological function of specific DEGs. Additionally, PPI network analysis revealed potential hub proteins and protein modules involved in the development of OM after *S. aureus* infection. Compared with healthy controls, 209 SARGs showed consistent expression trends in patients infected with *S. aureus* and even after grouping, which may imply a robust transcriptional signature in *S. aureus* infection. GO enrichment analysis showed that SARGs were primarily involved in innate immune and inflammatory response-related GO terms, as well as osteoclast differentiation, hematopoietic cell lineage, and immune-related pathways, which is consistent with previous studies (Ardura et al., 2009; Banchereau et al., 2012). Furthermore, 377 OMRGs were identified and appeared to have a potential role in the complex host-pathogen interaction in children with OM. However, there were significant differences between the results of the enrichment analysis of OMRGs and SARGs, which warrants more discussion.

Throughout the analysis of previous studies, the immune response was shown to be a crucial pathway of *S. aureus* infection. Previous investigators have identified an up-regulated pattern of genes pertaining to innate immunity concurrent with a down-regulated pattern of genes pertaining to adaptive immunity (Ardura et al., 2009; Banchereau et al., 2012). According to present results, we found that most of the top 15 hub proteins in PPI network of the SARGs were up-regulated, and several of them were structural components of the most recently described neutrophil extracellular traps (NETs), which suggested the importance of NETs in the immune mechanism. NETs have been described as a fundamental defense mechanism of innate immune (Brinkmann et al., 2004). Proteins with bactericidal activity, including ELANE, MPO, CTSG, and LL37, adhere to NETs and consequently destroy virulence factors (Delgado-Rizo et al., 2017).

The top 3 modules of PPI network were explored using MCODE. In our study, the PPI network of the SARGs seem to be highly clustered, which is characterized in the biological attributes, reflecting a high degree of modularization. Module 1 was an interesting module associated with specific functions, including ELANE, MPO, CAMP, CTSG, etc. Mutations in ELANE often cause cyclic and severe congenital neutropenia (Makaryan et al., 2017). MPO has been recently demonstrated to be associated with NETs production (Granger et al., 2017; Kenny et al., 2017). After uptake by neutrophils, *S. aureus* escapes from the MPO-mediated killing to enhance intracellular survival by secreting a unique proteinaceous MPO inhibitor (de Jong et al., 2017). CAMP, alias LL37, is also highly present in NETs, which can facilitates the formation of NETs (Neumann et al., 2014a) and stabilize NETs against bacterial nuclease degradation (Berends et al., 2010; Neumann et al., 2014b). CTSG and CAMP are both antimicrobial proteins with bacteriocidal activity against *S. aureus* (Bangalore et al., 1990; Neumann et al., 2014b). In addition, DEFA4 is an α-defensin that can stimulate IL-6 release in macrophages in a TLR4-independent way (Vandenbroucke et al., 2014). Based on studies mentioned above, we speculated that NETs may be critical for the immune response to the process of *S. aureus* infection.

However, the pathogenesis and progression of OM remain unclear. In this study, after we identified the SARGs of *S. aureus* infection, those named OMRGs, which were differentially expressed after successively developing into OM, were further investigated by analysis on the involving GO enrichments and pathways. Specifically, it is found that the OMRGs were significantly enriched in three pathways, namely alcoholism, systemic lupus erythematosus, and proteoglycans in cancer, and they may have a profound implication for the pathological responses of OM. GO functional analysis of the OMRGs were enriched in transmembrane signaling receptor and calcium channel activity, cilium morphogenesis, chromatin silencing, even multicellular organism development. Several key proteins, including PHLPP2 and EGF, were hub nodes in PPI network of the OMRGs.

PHLPP2, a member of the PH-domain leucine-rich repeat protein phosphatase (PHLPP) family, has been proved as a potential candidate biomarker which suppresses tumorigenesis and metastasis (O'Neill et al., 2013). PHLPP1α and PHLPP1β were identified as another two forms of PHLPP isozymes besides PHLPP2. PHLPP catalyzes the dephosphorylation of a group of kinases, such as Akt, PKC, and S6 kinase, as well as activates the pro-apoptotic kinase Mst1, thereby inhibiting cellular proliferation and inducing apoptosis (Nitsche et al., 2012; O'Neill et al., 2013). A series of studies confirmed that PHLPP1 controls chondrogenesis (Bradley et al., 2015) and *Phlpp1* deficiency protects against osteoarthritis progression (Bradley et al., 2016). Kang et al. demonstrated that PHLPP2 acts as an important mediator in the differentiation process of T follicular helper cells (Kang et al., 2013). Agarwal et al. indicated that PHLPP2 decreases the activation of NF-κB (Agarwal et al., 2014). Little is known, however, regarding the relationship between PHLPP2 and the skeletal system disorders. Therefore, more experimental verification is still needed to confirm our results.

The family of EGF receptors and their ligands, also known as ERBB family, have also been extensively explored for their roles in oncogenic function (Lu and Kang, 2010). EGF has been shown to play a crucial role in angiogenesis (Miyake et al., 2013; Lequoy et al., 2016) and executes its function through its involvement in binding to the four transmembrane tyrosine kinase receptors, including EGFR, HER2, HER3, and HER4. Because EGFR is mutated or overexpressed in numerous human cancers, it has become a major prognostic marker and therapeutic target (Herbst et al., 2004). Activated EGFR triggers serial activation of intracellular signaling, including PI3 kinase, MAP kinase and STAT pathways. Additionally, activation of STAT3 in response to IL-6 is prolonged in an EGFR dependent manner (Wang et al., 2013). More importantly, there is accumulating evidence indicating that EGF family might regulate the crosstalk between osteoclasts, osteoblasts and endothelial cells, which is essential for bone homeostasis (Yi et al., 2008; Zhang et al., 2011; Martín-Saavedra et al., 2017). Considering this, targeting EGF family may provide a novel strategy for OM therapy.

In conclusions, we identified 209 SARGs and 377 OMRGs in *S. aureus* infection and developing into OM, respectively. Our work suggested that NETs may be critical for the immune response to the process of *S. aureus* infection. In addition, the interaction between *S. aureus* and osteoblasts is a determining factor in the development of OM in bone tissue. And as such we speculate that the effect may be implemented by the OMRGs, including PHLPP2 and EGF, as well as three pathways (alcoholism, systemic lupus erythematosus, and proteoglycans in cancer) through which the OMRGs may be response to OM. The DEGs in our study may contribute to exploration of the underlying mechanisms of OM. As extension of this work, it requires further research and prudent experimental validation before any clinical use.

## Data availability statement

The datasets GSE30119 analyzed for this study can be found in the Gene Expression Omnibus (GEO) (https://www.ncbi.nlm.nih.gov/geo/query/acc.cgi?acc=GSE30119). All DEGs, enrichment analysis and module analysis results generated for this study are included in the manuscript and the Supplementary Files.

## Author contributions

PC, YaH, and BY conceived and designed the research. PC organized the database. ZY, GD, and YiH analyzed the data. PC wrote the first draft of the manuscript. ZY, GD, YiH, SC, YaH, and BY wrote sections of the manuscript. All authors contributed to manuscript revision, read, and approved the submitted version.

### Conflict of interest statement

The authors declare that the research was conducted in the absence of any commercial or financial relationships that could be construed as a potential conflict of interest.

## Abbreviations

BP: biological process
CC: cellular component
Ctrl: healthy controls
DEGs: differentially expressed genes
GEO: Gene Expression Omnibus
GO: Gene Ontology
KEGG: Kyoto Encyclopedia of Genes and Genomes
MF: molecular function
NETs: neutrophil extracellular traps
OM: osteomyelitis
OFI: OM-free infection
OMI: OM infection
OMRGs: OM-related genes
PPI: protein–protein interaction
*S. aureus*: *Staphylococcus aureus*
SARGs: *S. aureus* infection-related genes
SI: *S. aureus* infection.
